# Increased Levels of Circulating and Tumor-Infiltrating Granulocytic Myeloid Cells in Colorectal Cancer Patients

**DOI:** 10.3389/fimmu.2016.00560

**Published:** 2016-12-08

**Authors:** Salman M. Toor, Azharuddin Sajid Syed Khaja, Haytham El Salhat, Omar Bekdache, Jihad Kanbar, Mohammed Jaloudi, Eyad Elkord

**Affiliations:** ^1^College of Medicine and Health Sciences, UAE University, Al Ain, United Arab Emirates; ^2^Cancer Research Center, Qatar Biomedical Research Institute, College of Science and Engineering, Hamad Bin Khalifa University, Qatar Foundation, Doha, Qatar; ^3^Oncology Department, Al Noor Hospital, Abu Dhabi, United Arab Emirates; ^4^Tawam Hospital, Al Ain, United Arab Emirates; ^5^Institute of Cancer Sciences, University of Manchester, Manchester, UK

**Keywords:** myeloid cells, neutrophils, colorectal cancer, circulation, tumor microenvironment

## Abstract

Increased levels of myeloid cells, especially myeloid-derived suppressor cells (MDSCs), have been reported to correlate with bad prognosis and reduced survival in cancer patients. However, limited data are available on their conclusive phenotypes and their correlation with clinical settings. The aim of this study was to investigate levels and phenotype of myeloid cells in peripheral blood and tumor microenvironment (TME) of colorectal cancer (CRC) patients, compared to blood from healthy donors (HDs) and paired, adjacent non-tumor colon tissue. Flow cytometric analysis was performed to examine the expression of different myeloid markers in fresh peripheral blood samples from CRC patients and HDs, and tissue-infiltrating immune cells from CRC patients. We found significantly higher levels of cells expressing myeloid markers and lacking the expression of major histocompatibility complex class II molecule HLA-DR in blood and tumor of CRC patients. Further analysis revealed that these cells were granulocytic and expressed Arginase 1 indicative of their suppressive phenotype. These expanded cells could be neutrophils or granulocytic MDSCs, and we refer to them as granulocytic myeloid cells (GMCs) due to the phenotypical and functional overlap between these cell subsets. Interestingly, the expansion of peripheral GMCs correlated with higher stage and histological grade of cancer, thereby suggesting their role in cancer progression. Furthermore, an increase in CD33^+^CD11b^+^HLA-DR^−^CD14^−^CD15^−^ immature myeloid cells was also observed in CRC tumor tissue. Our work shows that GMCs are expanded in circulation and TME of CRC patients, which provides further insights for developing immunotherapeutic approaches targeting these cell subsets to enhance antitumor immune and clinical responses.

## Introduction

Evasion of immune response has been proposed as an emerging hallmark of cancer. Remarkable increase of cancers in immune-compromised patients provided further insights into the relationship between immune suppression and increased susceptibility to cancers (1, 2). Therefore, studies focusing on mechanisms involving host immune suppression have attracted great interest in recent years. Myeloid-derived suppressor cells (MDSCs) and T regulatory cells (Tregs) have been identified as key mediators in the negative regulation of immune responses in tumor microenvironment (TME). Several studies showed that elevated levels of immunosuppressive cells in periphery, tumor-draining lymph node, and TME correlates with worse prognosis and tumor progression in various cancers (3).

Myeloid-derived suppressor cells are a heterogeneous population of myeloid origin, which exhibit a potent immunosuppressive activity against T-cell responses (4). Cells of myeloid origin were first described in cancer patients as natural suppressor cells (5). MDSCs are expanded in different pathophysiological conditions including cancers (6). The terminal differentiation of immature myeloid cells (IMCs) is halted at varying stages of maturation and differentiation giving rise to a morphological mixture of granulocytic and monocytic cells, which are immunosuppressive in nature (7).

Myeloid-derived suppressor cells are commonly identified as cells expressing common myeloid markers CD33 and CD11b, but lack the expression of markers for mature myeloid and lymphoid cells and the major histocompatibility complex (MHC) class II molecule HLA-DR (7). Human MDSCs are broadly classified into monocytic (M) and granulocytic (G) or polymorphonuclear (PMN) subset based on the expression of CD14 and CD15 markers (8). Monocytic MDSCs (M-MDSCs) are mostly CD33^+^CD11b^+^CD14^+^HLA-DR^−/low^, whereas granulocytic MDSCs (G-MDSCs) are CD33^+^CD11b^+^HLA-DR^−/low^CD14^−^CD15^+^ (9, 10). A third population of MDSCs has also been described as immature or early stage MDSCs, lacking CD14, CD15, and HLA-DR (11). Surface markers of G-MDSC including CD11b, CD33, and CD15 are also well-established markers for mature neutrophil granulocytes. Neutrophils and G-MDSC share phenotypical properties and functional characteristics. It is challenging to distinguish between neutrophils and G-MDSC, and currently, there is no accepted consensus on exclusive markers to differentiate them. Pillay et al., however, suggested that G-MDSCs could be a phenotypic subset of neutrophils with functional heterogeneity (12).

Several studies have shown that levels of neutrophils and MDSCs are increased in peripheral blood and TME in various cancers and exhibit tumor-specific immunosuppression and tumor-promoting effects (13–15). However, neutrophils have been shown to exhibit a dual role in tumor development by either promoting the growth, invasion, and metastasis of tumor or by exerting tumoricidal activity through secretion of antitumoral factors or by stimulating T-cell responses and antitumor immunity (16, 17). Elevated neutrophil to lymphocyte ratio is frequently reported in cancer patients and is used as a predictive and prognostic factor in various human cancers (15). In relation to their immunosuppressive role, neutrophils and MDSCs utilize various mechanisms, which involve production of Arginase 1 (ARG1), inducible nitric oxide synthase, and reactive oxygen species (18, 19). These cells have also been shown to play important roles in promoting growth of various cancers including colorectal cancer (CRC) (20).

Colorectal cancer is the third most common cancer worldwide and is responsible for around 0.5 million deaths per year (21, 22). It is an inflammatory cancer, often closely related to inflammatory bowel disease, and inflammation is recognized as a key factor in the pathogenesis of both sporadic and hereditary CRC (23). The disruption of myelopoiesis and hemopoiesis are recognized as the key immune mechanisms involved in inflammation-related tumorigenesis, which result in the accumulation of MDSCs (24). Although higher levels of circulating or tumor-infiltrating MDSCs have been reported in CRC patients (25, 26), there is not enough information regarding the phenotypical characterization of these cells. Therefore, studies identifying and characterizing various subtypes of MDSCs in CRC patients are warranted. In the present study, we compared levels of circulating myeloid cells between healthy donors (HDs) and CRC patients and investigated ARG1 expression, indicative of their suppressive phenotype. We found significant increase in the levels of different subsets of myeloid cells in peripheral blood of CRC patients compared with HDs. We also found that higher levels of myeloid cells of granulocytic and immature phenotypes accumulated in tumor tissue (TT) compared with adjacent non-tumor colon tissue. Our findings identify the expansion of granulocytic myeloid cells (GMCs) in circulation and TME of CRC patients and provide insights for immunotherapeutic strategies targeting these cells in CRC patients.

## Materials and Methods

### Patients and Healthy Donors

Peripheral blood samples were collected in heparinized tubes (200 IU) from patients with CRC (*n* = 21) at Tawam Hospital, Al Ain, UAE and Al Noor Hospital, Abu Dhabi, UAE. Additionally, tumor tissues (TT) and paired, adjacent non-tumor colon tissues (NT) were collected from patients who underwent surgery (*n* = 9). Peripheral blood samples were also collected from HDs (*n* = 21) as controls. None of the patients included in this study received any treatment prior to surgery. Ethical approval was obtained from Al Ain Medical District Human Research Ethics Committee and written consent forms were signed by all patients and donors prior to collection of samples. Table 1 shows clinical and pathological characteristics of all participating individuals.

**Table 1:** **Characteristic features of study populations**.

	HD	CRC
Number	21	21 (9)^b^
Age (median)	30 (19–51)^a^	46 (32–74)^a^
Gender (male:female)	9:12	13:8
**TNM stage**		
I		2 (1)^b^
II		7 (3)^b^
III		11 (4)^b^
IV		1 (1)^b^
Tumor size (cm)		4.2 (0.2–10)^a^
Lymph node invasion		11
Liver metastasis		1
**Histological grade**		
Well/moderate		17
Poor/undifferentiated		4

*HD, healthy donor; CRC, colorectal cancer*.

*^a^Data shown represent median (range)*.

*^b^Samples taken from patients for investigating tissue-infiltrating immune cells*.

### Enzyme Disaggregation of Tumor and Normal Tissues for Cell Isolation

Fresh colorectal TT and NT were collected in cold RPMI-1640 media. Enzyme disaggregation (ED) of tissue was performed on a rollover mixer at 37°C for 60 min. Briefly, tissues were first washed with phosphate-buffered saline (PBS) and then mechanically cut into small pieces (around 2–4 mm) using a surgical scalpel. Samples were then suspended into RPMI-1640 with 1% penicillin/streptomycin and enzyme cocktail, consisting of 1 mg/ml collagenase, 100 μg/ml hyaluronidase type V, and 30 IU/ml of deoxyribonuclease I (all from Sigma-Aldrich, UK). The cell suspension was then passed through a 100 μm BD Falcon cell strainer (BD Biosciences, Oxford, UK) to remove debris and aggregates. Cells were then washed with RPMI-1640 and resuspended in RPMI-1640 enriched with 10% FCS and 1% penicillin/streptomycin.

### Staining of Whole Blood and Tissue-Infiltrating Immune Cells for Flow Cytometric Analyses

Two hundred microliters of blood from each sample was used for whole blood staining for different MDSC markers; 100 μl was used as non-stained control and 100 μl was stained for each sample. Mouse anti-human CD33-APC (Clone WM53), mouse anti-human CD11b-APC-Cy7 (Clone ICRF44), mouse anti-human HLA-DR-PE (Clone G46-6), mouse anti-human CD14-PerCP-Cy5.5 (Clone M5E2), and mouse anti-human CD15-PE-Cy7 (Clone HI98) antibodies were added to the stained sample. All antibodies were purchased from BD Biosciences. Tubes were incubated at 4°C for 25 min. RBC lysis buffer (BD FACS lysing solution) was then added to each tube and incubated in dark for 5 min. After washing the samples twice with PBS, cells were fixed and permeabilized using Fixation/Permeabilization Buffer (eBioscience, San Diego, CA, USA) and incubated at 4°C for 45 min after thorough vortexing. Samples were then washed twice with permeabilization wash buffer (eBioscience) and stained with sheep anti-human/mouse ARG1-FITC antibody (R&D Systems, Minneapolis, MN, USA) for intracellular staining by incubating at 4°C for 25 min, followed by two washes with wash buffer (eBioscience). The cell pellet was resuspended in 300 μl of flow cytometry staining buffer (eBioscience) to analyze on BD FACS Canto II flow cytometer (BD Bioscience, USA).

For staining immune cells extracted by ED, cells were first blocked for Fc receptor using FcR Blocker (Miltenyi Biotec, Bergisch Gladbach, Germany). 7AAD viability dye (eBioscience) was added, followed by staining with mouse anti-human CD11b-APC-Cy7 (BD Biosciences), mouse anti-human CD33-FITC (Clone P67.6, BioLegend, San Diego, CA, USA), mouse anti-human HLA-DR-PE (BD Biosciences), CD14-PE-Cy7 (Clone 61D3, eBioscience), and mouse anti-human CD15-APC (Clone HI98, BioLegend). After incubation at 4°C, samples were washed twice with PBS and the pellet was resuspended in 300 μl flow cytometry staining buffer (eBioscience) for analyzing on BD FACS Canto II flow cytometer. Flow cytometric data were analyzed on BD FACSuite software (BD Biosciences).

### Statistical Analyses

Statistical analyses were performed using GraphPad Prism 5.0 software (GraphPad Software, USA). Paired/Wilcoxon matched-pairs signed rank test or unpaired/Mann–Whitney tests were used to examine the differences within groups or between groups, respectively. A *p* value of ≤0.05 was considered statistically significant. The data are presented as means ± SEM. Levels of cells were measured as relative percentages or calculated percentages from parent population(s). For statistical analyses, staining of 20 peripheral blood samples from CRC patients was used.

## Results

### CD33^+^CD11b^+^HLA-DR^−/low^ Myeloid Cells Are Expanded in Peripheral Blood of CRC Patients, Compared with Healthy Donors

**Figure 1:**
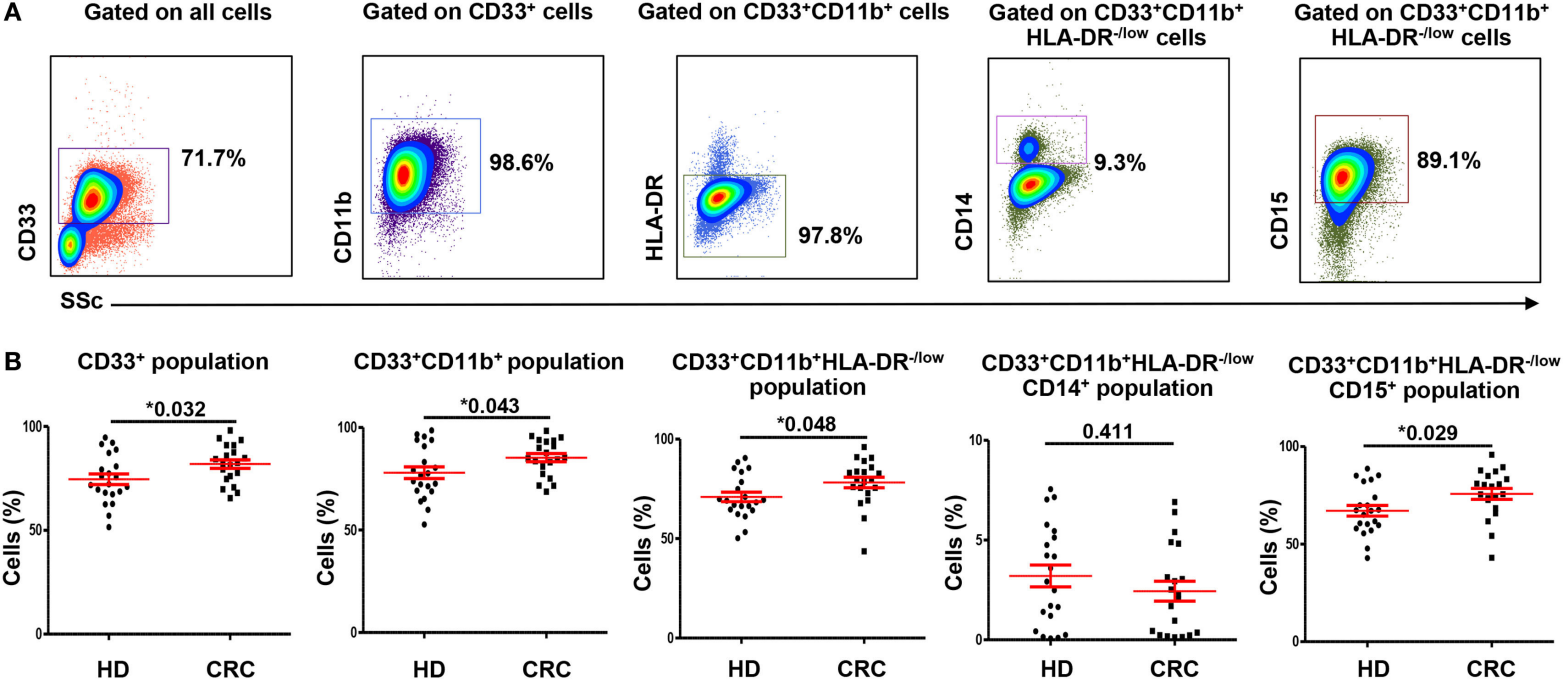
**Gating strategy of myeloid cells and comparisons of levels of different subsets of circulating myeloid cells between HDs and CRC patients**. Representative flow cytometric plots showing the gating strategy used to identify myeloid cells in peripheral blood of HDs and CRC patients **(A)**. CD33^+^ cells were gated first from all cells, followed by gating CD11b^+^ cells and HLA-DR^−/low^ cells from parent populations. MMCs were then identified as CD14^+^ cells, while GMCs were identified based on the expression of CD15. Fresh whole blood from 21 HDs and 20 CRC patients was stained for these markers. **(B)** Shows scatter plots of the means of calculated percentages ± SEM of each myeloid subpopulation including CD33^+^ cells, CD33^+^CD11b^+^ cells, CD33^+^CD11b^+^HLA-DR^−/low^ cells, CD33^+^CD11b^+^HLA-DR^−/low^CD14^+^ cells, and CD33^+^CD11b^+^ HLA-DR^−/low^CD15^+^ cells.

We investigated levels and nature of myeloid cells in circulation and TME of CRC patients using flow cytometric analysis of different markers. Flow cytometric plots of the gating strategy used to identify myeloid cells are shown in Figure 1A. Fluorescence minus one (FMO) controls were used for all markers to identify positive populations (Figures S1A–E in Supplementary Material). Scatter plots of the means of calculated percentages ±SEM of each myeloid subpopulation are shown in Figure 1B. The frequency of each population was calculated out of the parent population. The relative percentages of each population were multiplied by relative percentage of its parent population and the resulting number was presented as calculated percentage. We found that CRC patients had significantly higher levels of CD33^+^ cells compared with HDs (CRC: 85.3 ± 2.0% vs. HD: 78 ± 2.9%; Figure 1B). Further analysis showed a similar trend in the levels of CD33^+^CD11b^+^ cells; CRC patients showed significantly higher levels of CD33^+^CD11b^+^ subsets compared with HDs (CRC: 81.9 ± 2.1% vs. HD: 74.6 ± 2.6%; Figure 1B). MDSCs are known to lack the expression of HLA-DR (27, 28); therefore, we investigated expression of HLA-DR within CD33^+^CD11b^+^ subsets. Levels of circulating CD33^+^CD11b^+^HLA-DR^−/low^ subsets in CRC patients were also significantly higher compared with HDs as shown in Figure 1B (CRC: 78.2 ± 2.7% and HD: 70.9 ± 2.4%).

### The Expanded Myeloid Cells in Peripheral Blood of CRC Patients Are Granulocytic

To find out if the expanded circulating myeloid cells in CRC patients are granulocytic or monocytic, we analyzed expression of CD15 and CD14 within the CD33^+^CD11b^+^HLA-DR^−/low^ subsets. CRC patients showed significantly higher levels of circulating CD33^+^CD11b^+^HLA-DR^−/low^CD15^+^ cells compared with HD (CRC: 75.8 ± 2.8%, HD: 67.1 ± 2.7%; Figure 1B). These cells could be neutrophil granulocytes or G-MDSCs (12); however, due to lack of conclusive evidence on their suppressive ability, we referred to them as GMCs. We also investigated levels of CD14^+^ cells within the CD33^+^CD11b^+^HLA-DR^−/low^ populations in the two study groups. There was no significant difference in levels of CD33^+^CD11b^+^HLA-DR^−/low^CD14^+^ monocytic myeloid cells (MMCs) in CRC patients compared to HDs (CRC: 2.4 ± 0.5%; HD: 3.2 ± 0.5%; Figure 1B). The overall levels of MMCs were significantly lower than GMCs in the two study groups (*p* < 0.001) (Figure 1B). We also investigated the levels of IMCs subset, which lack CD14 and CD15 expression, but did not find any difference in level of these cells between the two study groups (data not shown).

### ARG1 Expression in Myeloid Cell Subsets

**Figure 2:**
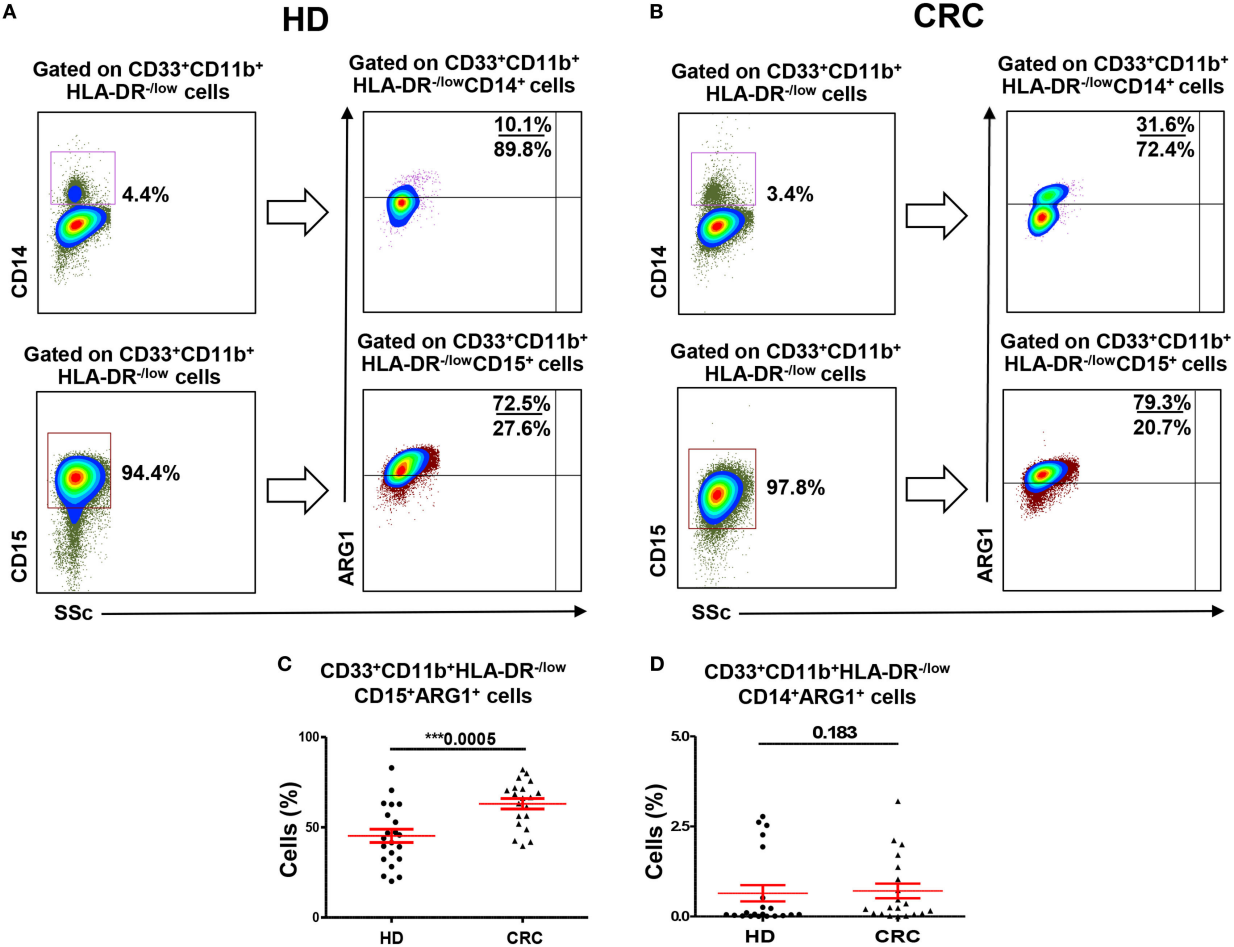
**Arginase 1 expression by MMCs and GMCs in peripheral blood of HDs and CRC patients**. Fresh blood from HD (*n* = 21) and CRC patients (*n* = 20) was stained for different MDSC markers to identify MMCs and GMCs cells, followed by intracellular staining for the expression of ARG1. Representative flow cytometric plots showing expression of ARG1 by CD33^+^CD11b^+^HLA-DR^−/low^CD14^+^ MMCs and CD33^+^CD11b^+^HLA-DR^−/low^CD15^+^ GMCs cells in a HD **(A)** and a CRC patient **(B)**. Scatter plots comparing calculated percentages ± SEM of ARG1 level in CD33^+^CD11b^+^HLA-DR^−/low^CD15^+^ **(C)** and CD33^+^CD11b^+^HLA-DR^−/low^CD14^+^ **(D)** subsets.

Myeloid-derived suppressor cells express high levels of ARG1, which assists in their characteristic attribute of T-cell suppression (6). We examined expression of ARG1 by both MMCs and GMCs. Figures 2A,B show flow cytometric plots of ARG1 expression by CD33^+^CD11b^+^HLA-DR^−/low^CD14^+^ MMCs and CD33^+^CD11b^+^HLA-DR^−/low^CD15^+^ GMCs in a HD and CRC patient, respectively. Flow cytometric plots for FMO for ARG1 are shown in Figure S1F in Supplementary Material. Both subsets expressed different levels of ARG1 (Figure 2). Levels of ARG1-expressing GMCs were significantly higher in peripheral blood of CRC patients than HDs (CRC: 63.1 ± 2.9% vs. HD: 45.3 ± 3.7%; Figure 2C). There was no significant difference in levels of ARG1-expressing MMCs in the two study groups (CRC: 0.7 ± 0.2, HD: 0.6 ± 0.2; Figure 2D). In line with our previous study in pancreatic cancer patients (29), GMCs expressed significantly higher levels of ARG1 than MMCs (Figures 2C,D).

### Tumor-Associated GMCs and IMCs Are Expanded in Colorectal Tumor Tissue, Compared with Normal Colon Tissue

**Figure 3:**
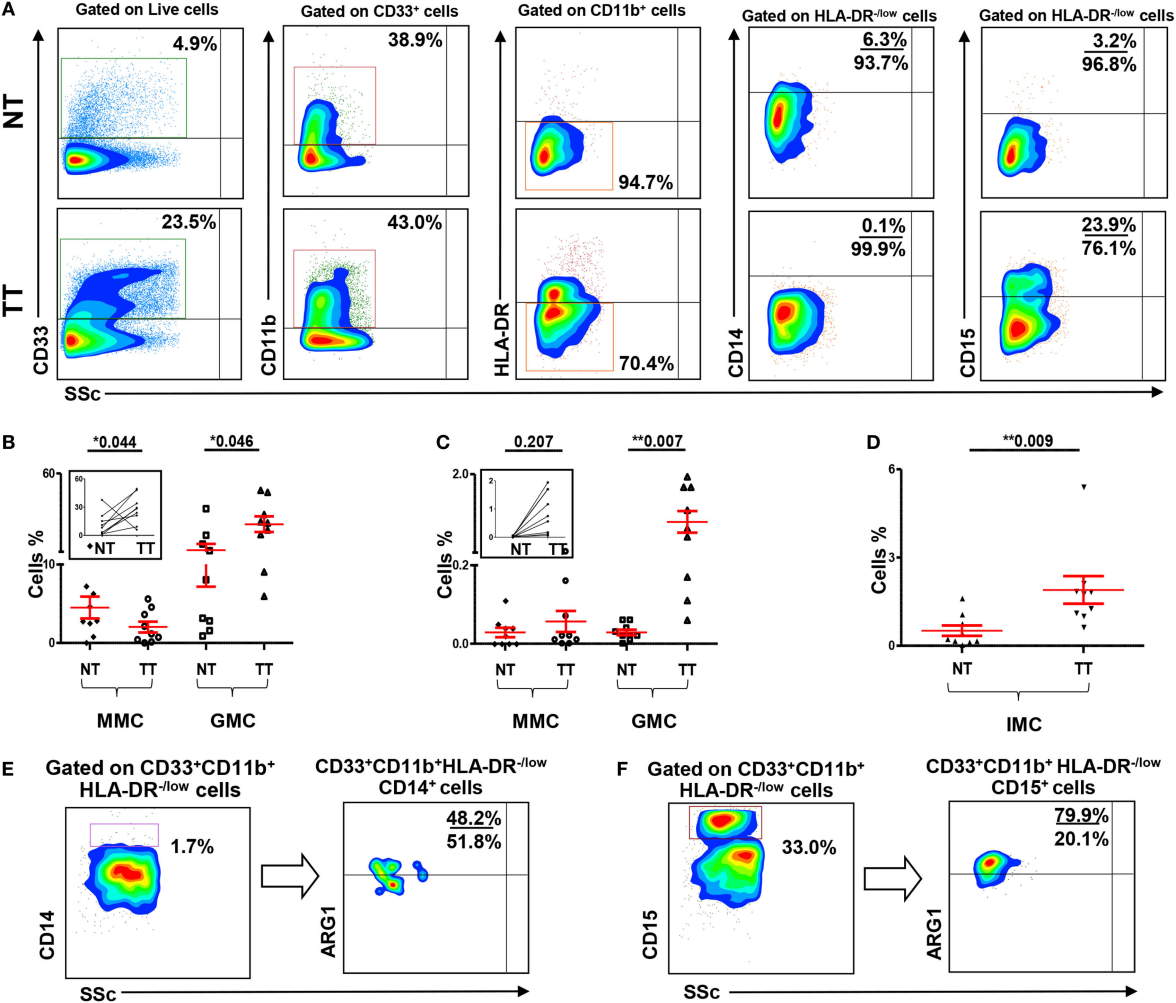
**Myeloid cells and arginase 1 expression in tissue-infiltrating immune cells**. **(A)** Representative flow cytometric plots showing levels of different subsets of myeloid cells in normal tissue (NT) and tumor tissue (TT) of nine CRC patients. Doublets were excluded and live cells were gated. The frequency of each population was calculated out of the parent population. Scatter plots showing mean of relative percentages ± SEM **(B)**, and calculated percentages ± SEM, calculated by multiplying the relative percentages of each subpopulation with their respective parent populations, of CD33^+^CD11b^+^HLA-DR^−/low^ CD14^+^ MMCs and CD33^+^CD11b^+^HLA-DR^−/low^ CD15^+^ GMCs in NT and TT of CRC patients **(C)**; inset showing the expansion of GMCs in TT compared with adjacent NT. **(D)** Means of calculated percentage ± SEM of CD33^+^CD11b^+^HLA-DR^−^CD14^−^CD15^−^ IMCs in NT and TT. Representative flow cytometric plots show the expression of ARG1 by tumor-infiltrating CD33^+^CD11b^+^HLA-DR^−/low^CD14^+^ MMCs **(E)** and CD33^+^CD11b^+^HLA-DR^−/low^CD15^+^ GMCs **(F)** in tumor tissue of CRC patients.

A major focus of this study was to investigate the nature and levels of myeloid cells in TME. We examined levels of tumor-infiltrating myeloid cells in CRC TTs and compared with NT from each patient (Figure 3A). Viable cells were first gated using 7AAD viability dye, followed by gating based on expression of myeloid cell markers. We found significant increase in levels of myeloid cells in TT compared with NT from nine CRC patients. There was an increase in CD33^+^, CD33^+^CD11b^+^, and CD33^+^CD11b^+^HLA-DR^−/low^ subsets in TT compared with NT in CRC patients (Figure 3A).

When further investigated, if these myeloid cells are of monocytic or granulocytic nature, we found that, similar to the results obtained in peripheral blood, this expansion was in CD33^+^CD11b^+^HLA-DR^−/low^CD15^+^ GMCs. There was a significant increase in the relative percentage of CD15^+^ GMCs in TT compared with NT (NT: 11.2 ± 4.0 vs. TT: 27.6 ± 5.0; Figure 3B). This difference was further confirmed when the numbers were calculated based on the percentage of parent population; there was a significant increase in the calculated percentages of CD15^+^ cells within CD33^+^CD11b^+^HLA-DR^−/low^ populations in TT compared with adjacent NT (NT: 0.03% vs. TT: 0.9%; Figure 3C). In contrast, there was a significant reduction in relative percentage, but not calculated percentage, of CD33^+^CD11b^+^HLA-DR^−/low^CD14^+^ MMCs in TT compared with NT (NT: 4.5 ± 1.4 vs. TT: 2.0 ± 0.7; Figures 3B,C). Furthermore, similar to peripheral blood, levels of GMCs were significantly higher than MMCs in both NTs and TTs (Figures 3B,C). There was also a significant increase in calculated percentage of CD33^+^CD11b^+^HLA-DR^−^CD14^−^CD15^−^ (IMCs) in TT compared with NT (Figure 3D).

We investigated expression of ARG1 by monocytic and granulocytic populations in cells isolated from NT and TT of three CRC patients. Both tumor-infiltrating CD33^+^CD11b^+^HLA-DR^−/low^CD14^+^ and CD33^+^CD11b^+^HLA-DR^−/low^CD15^+^ cells expressed ARG1. Representative plots showing expression of ARG1 in these cell subsets are shown in Figures 3E,F. However, sufficient data were not collected for statistical analysis due to limited number of samples.

### Circulating ARG1-Expressing GMCs Are Expanded in CRC Patients with Regional/Distant Metastases and in Patients with Poorly Differentiated Tumors

**Figure 4:**
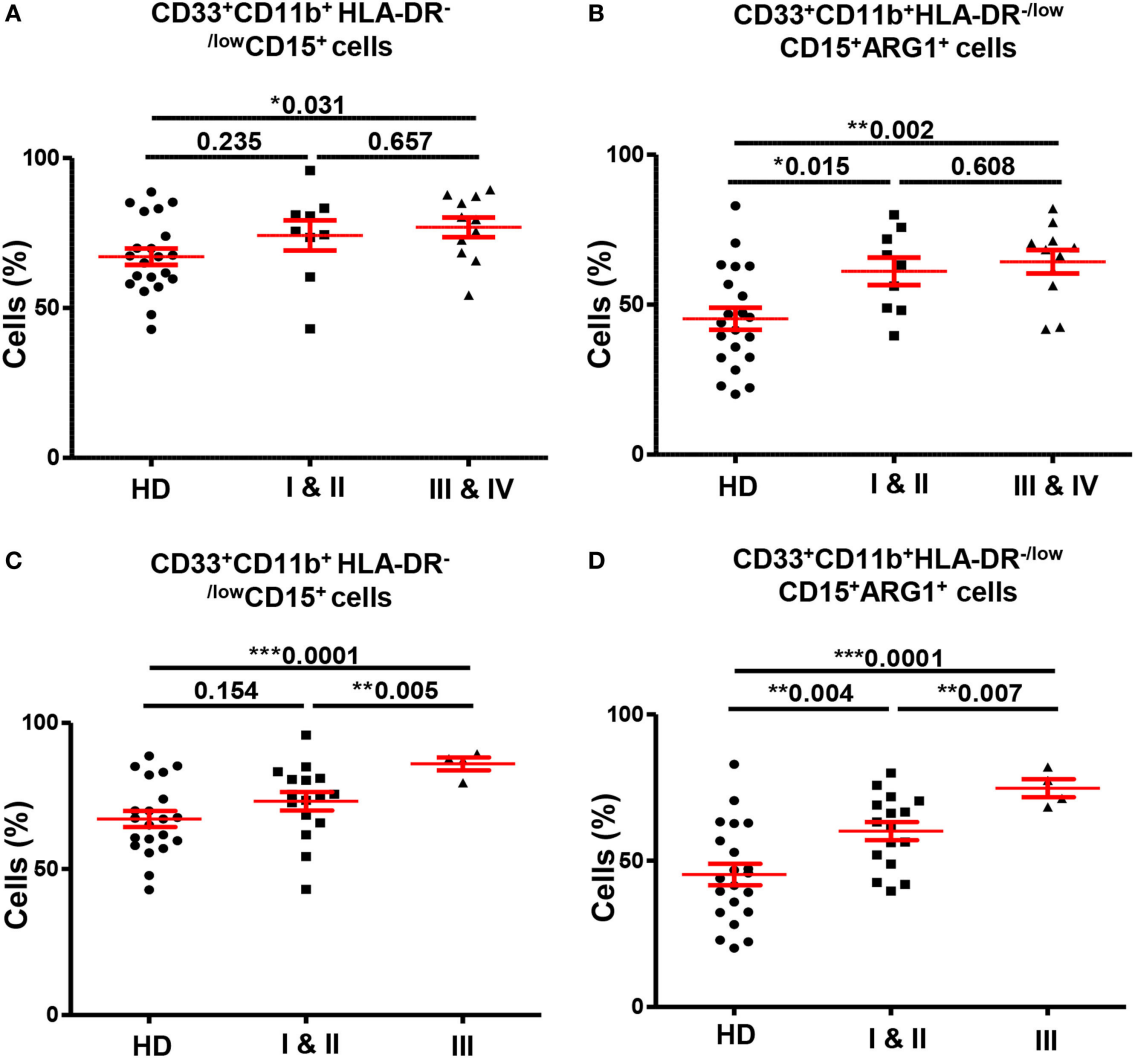
**Comparisons of levels of circulating GMCs between HDs and different TNM stages and histological grades of colorectal cancer**. Scatter plots comparing calculated percentages ± SEM of CD33^+^CD11b^+^HLA-DR^−/low^CD15^+^ GMCs **(A)** and their ARG1 level **(B)** between HDs and different TNM stages of CRC patients. Scatter plots comparing calculated percentages ± SEM of CD33^+^CD11b^+^HLA-DR^−/low^CD15^+^ GMCs **(C)** and their ARG1 level **(D)** between HDs and different histological grades of CRC patients.

Next, we investigated if the expansion of GMCs in CRC patients could potentially correlate with tumor stage or histological grade. In peripheral blood analysis, patients were divided into two groups according to TNM stage; patients with localized disease (stages I and II; *n* = 9) and patients with regional lymph node or distant metastases (stages III and IV; *n* = 11). We found significantly higher levels of circulating GMCs in patients with regional/distant metastases compared with HDs, and these cells expressed higher levels of ARG1 (Figures 4A,B). Although there was no difference in GMCs levels between HDs and patients with localized CRC (Figure 4A), GMCs in these patients expressed significantly higher levels of ARG1 compared with HDs (Figure 4B). There were no differences in ARG1-expressing myeloid cells when compared between patients with different tumor stages. Furthermore, there was no significant difference in MMCs levels between patients with different tumor stages and HDs (data not shown).

We also found significant differences in levels of circulating myeloid cells between HDs and CRC patients when categorized based on histological grade of differentiation of tumor cells (Figures 4C,D). Patients with histological grade I and II cancers had well to moderately defined tumor cells and are grouped as “low-grade tumors”; while grade III patients presented with poorly or undifferentiated tumor cells and are referred as “high-grade tumors”. CRC patients with high-grade tumors (*n* = 4) had significantly higher levels of circulating CD33^+^, CD33^+^CD11b^+^, and CD33^+^CD11b^+^HLA-DR^−/low^ cells compared with HDs (*n* = 21) and patients with low-grade tumors (*n* = 16). The expansion of CD33^+^CD11b^+^HLA-DR^−/low^ cells was due to increase in the granulocytic CD15^+^ subset; high-grade CRC patients had significantly elevated levels of GMCs than HDs (high-grade: 86.0 ± 2.2 vs. HDs: 67.1 ± 2.7; Figure 4C) and low-grade CRC patients (low-grade: 73.2 ± 3.2; Figure 4C). Furthermore, these cells expressed high levels of ARG1 (high-grade 74.8 ± 3.1; HDs: 45.26 ± 3.7; low-grade: 60.1 ± 3.1; Figure 4D). There was no difference in expression levels of CD33^+^CD11b^+^HLA-DR^−/low^CD15^+^ cells between patients with low-grade tumors and HDs (Figure 4C), but these cells expressed significantly higher levels of ARG1 in low-grade CRC patients compared with HDs (Figure 4D). There was no difference in the levels of MMCs and their ARG1 expression between the two patient subgroups and HDs (data not shown). For tumor-infiltrating immune cells, it was not possible to compare patients based on tumor stage or histological grade due to limited sample size.

## Discussion

In recent years, several studies have reported expansion of regulatory myeloid cells including neutrophils and MDSCs in peripheral blood and TME of different human cancers (9, 12, 15, 19). Human MDSCs consist of immature progenitor cells and mature cells of mononuclear or PMN phenotype. However, due to their heterogeneous nature, specific markers of human MDSCs are not well defined. It is also very difficult to discriminate between G-MDSCs and neutrophil granulocytes, which share phenotypical and functional characteristics. As a result, most of the studies have provided inconclusive evidence of their immunophenotypical nature. Moreover, a role of tumor-infiltrating myeloid cells in cancer patients is not well defined, and very few studies have reported their levels in peripheral blood and TME from the same cancer patients. In this study, untreated CRC patients with different tumor stages and histological grades were examined for the parallel analysis of myeloid cell levels in circulation and TME, and were compared with their levels in peripheral blood of HDs. As a control for their levels in TME, cells isolated from paired, adjacent non-tumor colon tissue were used from the same patients.

We used freshly drawn whole blood for analysis because various studies emphasized on the importance of sample handling and processing when monitoring levels of myeloid cells in circulation (10, 28, 30, 31). Florcken et al. did not find any differences in the expression levels of MDSC when analyzed in whole blood or PBMCs (31). However, Mandruzzato et al. suggested to monitor MDSC levels in whole blood instead of Ficoll-purified mononuclear cells as some MDSC subsets might not be detected if only PBMCs were examined (30). Fresh blood is preferred to prevent possible loss of GMCs and attenuation of cell surface markers due to Ficoll gradient separation (10).

We found significantly higher levels of circulating CD33^+^CD11b^+^ HLA-DR^−^ cells in CRC patients compared with HDs. This expansion is in accordance with previously reported findings (25, 26, 28). Additionally, we report that these expanded cells are granulocytic in nature. Studies on other cancers including cutaneous melanoma also reported the accumulation of circulating granulocytic MDSC like cells in patients compared to HDs (10, 32). Zhang et al., however, reported the expansion of IMCs lacking CD14 and CD15 in CRC (26). Furthermore, in contrast to monocytic MDSCs, which are commonly reported within different cancers, less evidence is available on levels of granulocytic MDSCs in cancers (33). Thus, our findings provide a further insight into myeloid cells in CRC.

Previous studies have investigated the functional activity of neutrophils and MDSCs through their suppression of T cells *in vitro* and expression of ARG1. T-cell suppression by myeloid cells is well documented in different cancers, such as gastric cancer (34), prostate cancer (35), breast cancer (28), and CRC (26). l-arginine is required for the proliferation and function of T cells and ARG1 results in the depletion of l-arginine in the microenvironment, thereby limiting its availability for T cells, which reduces their expression of CD3ζ chain, inhibit T-cell proliferation, and induce T-cell apoptosis (32, 36). It has previously been shown that production of ARG1 by mature myeloid cells in TME could inhibit antigen-specific T cell responses (37). Other studies have also demonstrated T-cell suppression by MDSCs through ARG1 (38). We found that the expanded circulating GMCs express ARG1 with higher mean fluorescence intensity (MFI) than MMCs (data not shown). Similar to peripheral blood, we found significant accumulation of tumor-infiltrating myeloid cells compared to adjacent NT, which were primarily granulocytic and expressed ARG1.

Circulating GMCs were significantly elevated in CRC patients with regional and distant metastases compared with HDs. However, there were no differences in GMCs levels between patients with different tumor stages. These results are in agreement with previous studies in CRC and cutaneous melanoma, which have also shown higher levels of MDSCs in metastatic cancers compared with control group (10, 26). There are studies, however, which reported significant differences in levels of myeloid cells between HDs and patients with low stage cancers, and between patients with different cancer stages (25, 28); this discrepancy could be due to the number of patients and type of cancer investigated.

Previous studies have highlighted the immunosuppressive functions of neutrophils and myeloid cells, which would facilitate tumors to evade local immune response, and a role of GMCs in invasion and metastases is emerging from ongoing research (11, 12, 19). Tumor cells secrete several soluble factors such as Interleukin 6 and GM-CSF, which influence bone marrow and help in expansion of myeloid cells. These cells accumulate in TME and produce factors such as VEGF, matrix metallopeptidase 9 (MMP9), and transforming growth factor beta (TGF-β), which support angiogenesis and tumor growth and eventually invasion and metastases of tumor cells (11, 15, 39). Thus, our results suggest a role of immunosuppressive GMCs in cancer invasion and metastases.

Grading of tumors depends upon histological differentiation of tumor cells compared to normal cells, and it represents how quickly a tumor can grow and spread. Histological grade has been shown to be stage-independent prognostic variable in CRC patients; high-grade CRC patients with poorly or undifferentiated cancer cells have worse disease prognosis compared to low-grade CRC patients with well or moderately differentiated tumor cells (40, 41). Our work shows that in high-grade CRCs, GMCs may play important role in tumor-mediated immune suppression and suggest their involvement in tumor progression.

In conclusion, our study shows that GMCs and IMCs are expanded in CRC patients. Importantly, expansion of GMCs correlated with tumor stage and histological grades, thereby identifying these cells as key players among others in CRC patients. Better understanding of their characteristics should aid in therapeutic strategies to target immunosuppressive pathways employed by tumors.

## Author Contributions

ST and AK performed experimental work and data analysis and wrote the manuscript. EE conceived the idea, designed the study, obtained fund, analyzed and interpreted data, and wrote and revised the manuscript. HS, OB, JK, and MJ contributed to sample collection, acquisition of patients’ clinical data, and revising the manuscript. All the authors were involved in the final approval of the manuscript.

## Conflict of Interest Statement

The authors declare that the research was conducted in the absence of any commercial or financial relationships that could be construed as a potential conflict of interest.
