# Supplementary figures and images for: Increased Levels of Circulating and Tumor-Infiltrating Granulocytic Myeloid Cells in Colorectal Cancer Patients
Source: Front Immunol. 2016 Dec 8;7:560. doi: 10.3389/fimmu.2016.00560 (PMC5143474; doi:10.3389/fimmu.2016.00560)

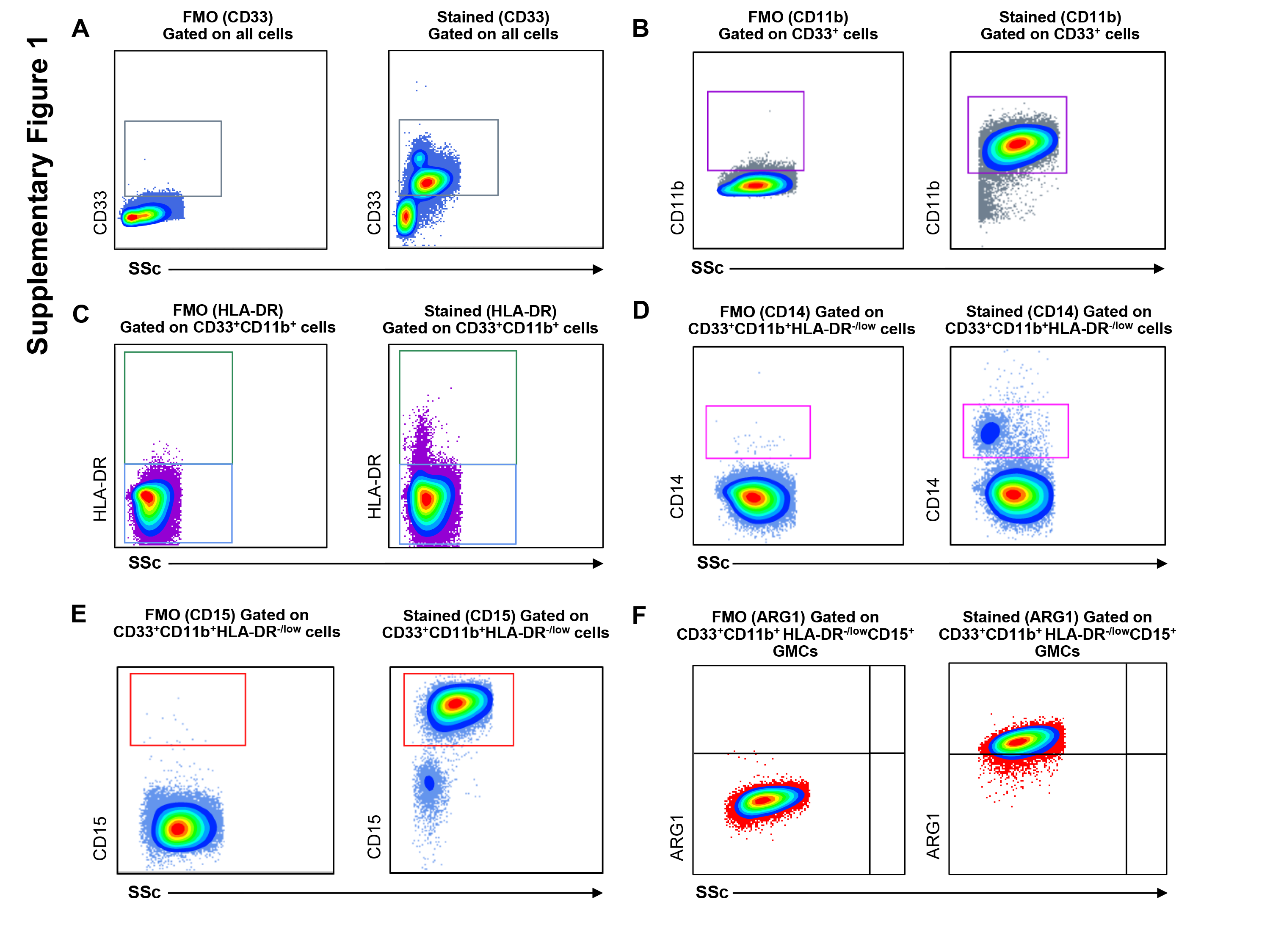

Supplement: Supplementary file 1 [file Image_1.TIF]
